# Environmental Drivers of West Nile Fever Epidemiology in Europe and Western Asia—A Review

**DOI:** 10.3390/ijerph10083543

**Published:** 2013-08-09

**Authors:** Shlomit Paz, Jan C. Semenza

**Affiliations:** 1 Department of Geography and Environmental Studies, University of Haifa, Mt. Carmel, Haifa 3498837, Israel; 2 European Centre for Disease Prevention and Control (ECDC), Tomtebodavägen 11A, Stockholm 17183, Sweden; E-Mail: Jan.Semenza@ecdc.europa.eu

**Keywords:** West Nile Virus, West Nile Fever, Europe, environmental drivers, epidemiology, climatic factors, climate change, bird migration, land use

## Abstract

Abiotic and biotic conditions are both important determinants of West Nile Fever (WNF) epidemiology. Ambient temperature plays an important role in the growth rates of vector populations, the interval between blood meals, viral replication rates and transmission of West Nile Virus (WNV). The contribution of precipitation is more complex and less well understood. In this paper we discuss impacts of climatic parameters (temperature, relative humidity, precipitation) and other environmental drivers (such as bird migration, land use) on WNV transmission in Europe. WNV recently became established in southeastern Europe, with a large outbreak in the summer of 2010 and recurrent outbreaks in 2011 and 2012. Abundant competent mosquito vectors, bridge vectors, infected (viremic) migrating and local (amplifying) birds are all important characteristics of WNV transmission. In addition, certain key climatic factors, such as increased ambient temperatures, and by extension climate change, may also favor WNF transmission, and they should be taken into account when evaluating the risk of disease spread in the coming years. Monitoring epidemic precursors of WNF, such as significant temperature deviations in high risk areas, could be used to trigger vector control programs and public education campaigns.

## 1. Introduction

West Nile virus (WNV) was first recognized in Uganda in 1937 when it was isolated from the blood of a woman inflicted with a mild febrile illness. WNV is an enveloped, single-strand RNA virus of the genus *Flavivirus*, family *Flaviviridae*, which includes several other human pathogens such as dengue, Japanese encephalitis and yellow fever viruses. WNV is transmitted between birds via mosquito vectors. The enzootic cycle is driven by continuous virus transmission to susceptible bird species through adult mosquito blood-meal feeding which results in virus amplification. Species from the genus *Culex* mosquitoes (family *Culicidae*) are the most important vectors [1]; however, it remains unclear how the composition of the mosquito community influences the timing and intensity of avian epizootics and human epidemics [2]. Phylogenetic analyses showed two distinct lineages of WNV strains (which themselves subdivide into several subclades or clusters), isolated in different geographic regions [3].

The transmission cycle involve wild birds as the principal hosts and mosquitoes, largely bird-feeding species, as the primary vectors. It exists in rural ecosystems as well as in urban areas where mosquitoes breed in organic-rich water in artificial containers [4,5,6,7,8,9,10,11]. In particular, low-lying places with poor drainage, urban catch basins, roadside ditches, sewage treatment lagoons, and manmade containers around houses provide good larval development sites for *Culex* spp. mosquitoes to deposit their eggs [10]. 

Most human infections occur in the summer or early fall [12]. West Nile Fever (WNF) is a potentially serious illness for humans and about one in 150 people infected with WNV will develop a severe illness with symptoms that may last several weeks. Neurological effects may be permanent. Up to twenty percent of the infected people have milder symptoms and approximately eighty percent of people who are infected with WNV will show no symptoms at all [13]. 

**Figure 1:**
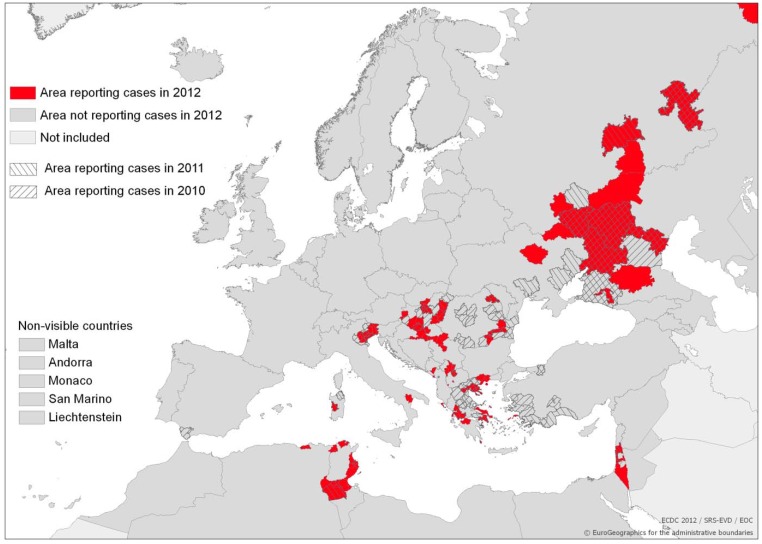
Reported cases of West Nile fever for the EU and neighboring countries. Transmission season 2010–2012; latest update: 30/11/2012. Source: © ECDC.

WNV is widely distributed throughout the tropical and temperate regions of the world [14] and is a vector-borne pathogen of global importance. It is now endemic in Africa, the Americas, Asia, Australia, Eurasia, and the Middle East, where it infects birds, humans, horses and other mammals. In Europe, WNV emerged in the Camargue area of southern France in the 1960s. Subsequently, European outbreaks in humans and horses have been documented in: Bucharest, Romania (1996–1997; 2003–2009), the Czech Republic (1997), Volgograd, Russia (1999), France (2000, 2003, 2004, 2006), Italy (1998, 2008, 2009), Hungary (2000–2009), Spain (2004) and Portugal (2004). In 2010, large outbreaks occurred in southeastern Europe in the Central Macedonia Region in northern Greece, as well as in Romania, Hungary, Italy and Spain, in Volgograd, Russia, Turkey and Israel (Table 1). These outbreaks were accompanied by infections in donkeys in Bulgaria and horses in Portugal, southern Italy, Greece and Morocco and showed no signs of abating in 2011 or 2012 (Figure 1; Table 1). 

**Table 1:** WNF cases in humans in Europe and bordering countries, 2010–2012. Source: ECDC database.

Country	Number of cases		
	2010	2011	2012
Russia	419	135	399
Greece	262	100	161
Israel	105	33	59
Romania	57	10	14
Turkey	47	3	0
Italy	6	14	50
Spain	2	0	0
Hungary	18	0	10
Ukraine	0	5	0
Albania	0	2	0
Macedonia	0	4	6
Tunisia	0	3	32
Serbia	0	0	67
Croatia	0	0	5
Kosovo	0	0	4
Palestinian Territory	0	0	2
**Total**	**1,016**	**309**	**810**

Phylogenetic studies on WNV strains isolated from regions of Greece, Italy, Spain, Portugal, France and Romania indicate that the virus has become established in Europe [15,16,17,18,19,20,21,22]. Yet, the mechanisms of virus introduction and spread in Europe are not fully understood [15]. The lack of WNF cases in humans in northern Europe, compared to southern Europe, may possibly be attributed to the feeding behavior of the predominant vector, *Cx. pipiens* as well as other factors, especially climate [23].

The aim of the current review is to discuss the significance of environmental drivers for WNF epidemiology in Europe and Western Asia and to suggest possible explanations for how WNV has become an endemic pathogen in many parts of the continent.

## 2. Environmental Drivers

Abiotic (physical features of the environment) and biotic (e.g., host abundance and diversity) conditions are both important determinants of WNF epidemiology [24]. The establishment and transmission of WNV is determined by a number of factors: the presence of susceptible avian hosts, infected (viremic) birds, and local (amplifying) birds; abundant competent mosquito vectors that feed on birds and bridge vectors that feed both on birds and humans/horses; and finally interactions of the pathogen and the vector with the biotic and abiotic environment. The epidemiology of the disease is also a function of a vector and vertebrate host population densities that facilitate WNV amplification among competent insect vectors and vertebrate hosts. The intensity of the transmission of WNV is related to the dynamics and interactions between the pathogen, vector, and vertebrate hosts [24,25,26]. 

Moreover, factors potentially influencing the maintenance (endemization) of WNV (e.g., weather conditions and land use characteristics) and those that affect its spread (migratory birds and short distance vector transportations) can also be distinguished.

### 2.1. Climatic Factors

Among other drivers, weather conditions and climatic factors were found to be direct abiotic factors that influence vector competence (their ability to acquire, maintain, and transmit the virus) for WNV. Moreover, the presence of favorable ecological habitats for vectors and hosts, with permissive weather conditions and their appropriate seasonal timing are crucial constituents for a successful transmission cycle [27,28].

#### 2.1.1. Ambient Temperature

Ambient temperature plays an important role in viral replication rates and transmission of WNV, affecting the length of extrinsic incubation, seasonal phenology of mosquito host populations and geographical variation in human case incidence [14,28,29,30,31]. Elevated ambient temperatures increase growth rates of vector populations [32], decrease the interval between blood meals, and accelerate the rate of virus evolution [28,30,33,34].

For instance, experimental study by Dohm and Turell [35] showed that WNV was recovered from most *Cx. pipiens* mosquitoes held exclusively at 26 °C but none of the mosquitoes held exclusively at lower temperatures had detectable infections. Dohm *et al*. [1] detected that in *Cx. pipiens* held at 30 °C, the virus was recovered from nearly all mosquitoes tested (as early as four days after the infectious blood meal) whereas for mosquitoes held at 18 °C, disseminated infections were not detected until 25 days after the infectious blood meal. In a laboratory experiment, Kilpatrick *et al*. [30] determined how temperature and time since feeding on infected blood affected the probability that *Cx. pipiens* would transmit two genotypes of WNV (NY99 and WN02). They found that the advantage of the WN02 genotype increased with the product of time and temperature (at selected days from four to forty days at 15 °C, 18 °C, and 22 °C and at half days from 0.5 to 3 for experiments at 32 °C) and concluded that the transmission of WNV accelerated sharply with increasing temperature. In a modeling study, Ruiz *et al.* [34] showed that temperature mediates the magnitude and timing of increased minimum infection rate within a season. The effect of increased temperature was especially strong within a week.

WNV is found in diverse sub-climatic regions (for example from Canada to the tropical zones of America) and can be transmitted under a variety of temperature regimens [14]. Although the virus is capable of replication across a wide range of temperatures, from 14 °C in poikilothermic mosquitoes [27] to 45 °C in febrile avian hosts [29], the replication cycle is completed more quickly in mosquitos at higher temperatures [36,37]. Indeed, a clear association was found between extreme heat and outbreak intensity in humans [1,27,38,39,40]. At the same time, it is important to note that extremely high temperatures begin to slow mosquito activity. For example, temperatures above 30 °C reduced larval survival of *Cx. tarsalis* [41] and slowed WNV growth in *Cx. Univittatus* [14].

Thus, climate change is also an important environmental driver for WNV [42,43,44]. This affects ambient temperature, which in turn affects WNV transmission. Indeed, global warming has been documented through many scientific measurements [45,46]. According to the United Nations Environment Programme (UNEP), climate change “is the major, overriding environmental issue of our time, and the single greatest challenge facing environmental regulators. It is a growing crisis with economic, health and safety, food production, security, and other dimensions” [47]. It also affects human health via pathways of varying complexity, scale and directness and with different timing. Similarly, impacts (both positive and negative) vary geographically as a function of the physical and environmental conditions as well as the vulnerability of the local human population [48,49,50,51].

In parts of Europe, climate change resulting in warmer conditions may facilitate the establishment of WNV in new areas through an expansion of range and seasonal abundance of vector species, and by directly increasing competence for transmission. It has been shown by Kilpatrick *et al*. that WNV temperature-transmission in mosquitoes accelerates nonlinearly with the extrinsic incubation temperature. Therefore, even slight increase of the ambient temperature may have a significant impact on the transmission [30].

A number of WNF outbreaks in Europe and neighboring countries have been associated with favorable environmental temperature conditions for mosquito proliferation [40,48,52]. A recent example was an outbreak in the summer of 2010, when southeastern Europe and Eurasia experienced an unprecedented upsurge in the number of human WNF cases (Figure 1). Most of the cases were reported between 26 June and 31 October, while the main outbreak occurred between the end of July and the end of September. A clear peak was observed between 23 August and 5 September. This outbreak was preceded by extremely hot spells: the period from the end of July to mid-August was extremely hot in Russia (deviations >9 °C above the 30 year mean average), in Romania and Turkey (>5 °C), and less so in Greece (>3 °C) [28]. This hot summer was a part of a continuing trend of a significant temperature increase in Europe. According to the World Meteorological Organization, this warming trend peaked this last decade ending in 2010, which was also one of the three hottest years ever recorded. During that summer, Eurasia endured exceptional heat waves while southeastern Europe experienced a record-setting sequence of consecutive hot nights [53]. Paz *et al*. [28] described climatic drivers of the 2010 outbreak in Eurasia (Figure 1; Table 1). Temperature was the most important driver: “colder” countries of more northern latitudes displayed strong statistically significant correlations between the number of WNF cases and temperature, with lags of up to four weeks from the onset of increased temperatures; in contrast, “warmer” and more southern countries presented correlations without such lags.

Reisen *et al*. [14] indicated that WNV tends to disperse into new areas during years with above-normal summer temperatures and that amplification during the following year have occurred in summers with above- or normal temperatures. Recent events in New York City, Israel and Europe illustrate that even if the summer subsequent to the first eruption that coincided with extreme heat is less hot, the disease can become endemic in the region [14,28,32,38]. Indeed, the WNF outbreaks in Europe and Eurasia during the summers of 2011 and 2012 occurred in most of the disease locations in 2010 (Figure 1; Table 1). It might therefore be reasonable to assume that increases in ambient temperature in light of climate change may contribute to the endemization process of WNF in Europe.

#### 2.1.2. Precipitation

Because the role of precipitation in WNV transmission is more indirect, impacting abiotic conditions and biotic factors [54], the scientific literature presents inconsistent results. Although the patterns of disease incidence can be influenced by the amount of precipitation, the response may change over large geographic regions, depending on differences in the ecology of mosquito vectors [55].

Above-average precipitation may lead to a higher abundance of mosquitoes, and also to increased potential for disease outbreaks [56], as was shown by Soverow *et al*. [57] who found positive associations between heavy rainfall (≥50 mm in a single day) and higher incidence of WNF in the United States. *Cx. pipiens* breeds in polluted, eutrophic waters. Heavy rainfall can flush the ditches and drainage channels used by *Culex* larvae [58] as was demonstrated experimentally by Koenraadt and Harrington [59] who showed that *Cx. pipiens* larvae were flushed out with longer rain exposure. In a study on WNV infection onset in horses in Europe during the spring-summer of 2010, the authors found that three infected sites—Trapany (Sicily, Italy), Campobasso (Molise, Italy), and Thessaloniki (Greece) were rainier than usual in July. These precipitations might have increased the standing water availability, an important breeding resource for mosquitoes. A rainfall increase in Gibraltar later in the season (during August) could explain the delayed appearance of the disease observed in this area [28].

On the other hand, below-average precipitation, such as drought, can also facilitate population outbreaks of some species of mosquitoes since the drying of wetlands disrupts the aquatic food-web interactions that limit larval mosquito populations [41]. When surface pools shrink during drying conditions, the remaining water grows more eutrophic and such environmental changes favor *Cx. pipiens* [58]. Moreover, drought leads to close contact of avian hosts and vector mosquitoes around remaining water sources. This facilitates the epizootic cycling and amplification of WNV within these populations [60]. A recent example was the WNF outbreak in Texas (USA) in the summer of 2012 that was attributed in part to drought conditions, which reduced water flow and created stagnant water pools ideal for breeding mosquitoes [61].

Uejio *et al*. [62] illustrated the rainfall pattern influences on WNV transmission from one year to the next. They found that the previous year’s precipitation is emerging as a driver of virus transmission in subtropical areas, while the effect is modified by geographic location. For example, in South African Highveld as well as west of the Mississippi River (excluding California), reverse associations were found while more precipitation in the previous year decreases the current year’s WNV transmission. 

Similarly, WNV infection in mosquitoes was negatively correlated with the previous year’s precipitation in research on WNV in *Culex* species in northeast Illinois [34]. The authors noted, however, that the mechanisms by which past weather conditions influence WNV transmission are still unclear.

In the tropics, the incidence should be greatest during the rainy season when mosquitoes are most abundant [12], but the published information on the epidemiology or ecology of WNV in the tropics is limited. While precipitation influences the emergence of mosquito populations in temperate climates [63], increased rainfall in a humid region may have little influence on the mosquito population if immature mosquito habitats are already abundant. However, mosquitoes in a more arid region, such as a Mediterranean climate type, could better propagate with increased precipitation as this may greatly increase the number of immature habitats, especially when heavy rainfall during the spring increases the standing water resources at the beginning of the hot season [32].

#### 2.1.3. Relative Humidity

Research into the linkage between relative humidity and WNV is limited. Significant positive correlations were found between relative humidity in the Tel-Aviv metropolis (Israel) and hospital admission dates of WNF patients [40]. A subsequent study detected correlations between WNF morbidity and weekly relative humidity in Europe [28]. Yet both studies noted that air temperature is a better predictor for increasing WNF cases than air humidity.

### 2.2. Landscape Features and Land-Use

Landscape features such as topography, soil moisture, surface water and general water quality, play important roles in the reproductive success of the vector species [64] and in virus endemization. Differences are known between species: while *Cx pipiens* is considered an urban species, and is especially successful in organic-rich water in artificial containers [65], *Cx. restuans* utilizes a wide range of habitats. The habitat of *Cx. quinquefasciatus* includes water with a high organic content often found in urban or peridomestic habitats, and these mosquitoes are known to bite indoors [66,67].

In Europe, circulation of WNV is mainly confined to two land-use patterns: first, the virus circulates in rural areas where wetlands serve as bird-nesting areas that provide ideal conditions for establishing endemic cycles of WNV. These ecosystems include river deltas and floodplain areas where bird-feeding mosquitoes (*Culex pipiens pipiens*, *Cx. modestus*, *Mansonia richiardii*) thrive and the bird-mosquito cycle propagates. Most WNF outbreaks in Europe have occurred in wetland areas such as the Rhone delta in southern France, the Volga delta in southern Russia, and the Danube delta in Romania [4]. The Camargue area is considered an endemic zone for WNV since the 2000s outbreak in southern France. This area along the Rhone river delta, presents diversified environments, consisting of dry areas irrigated by canals, ditches and wetlands. Geographic Information System and remote sensing technologies have suggested that arid shrubland, open water and woodlands are the major landscape categories associated with the risk of disease in horses in the wet zone. This may be explained by the fact that larval habitats are quiet permanent for mosquito species (e.g., *Cx. modestus*) in wet areas. In addition, woodlands, arid shrubland and open water are attractive biotopes for resting and nesting birds [68]. Another study in a wet zone in southern France analyzed the linkage between landscape categories and WNV circulation (equine serological data) and showed again that the same landscape features are the major landscape categories associated with the disease risk [69].

The second land-use pattern that influences the circulation of WNV exists in urban areas where mosquitoes feed both on birds and on humans and thus act as bridge vectors. Large human outbreaks were reported in Eurasia and Europe in densely inhabited urban areas: in Bucharest, Romania [70] Volgograd, Russia [43,71] and in Thessaloniki, Greece [72,73]. During the 1996–1997 outbreaks in Romania, flooded apartment buildings served as breeding grounds for mosquitoes. Apart from that case, research on urban parameters as potential drivers for WNV spreading in Europe is still limited. For comparison, large epidemics were confirmed in urban counties in the USA, with the insight that urbanization is becoming a significant risk factor for WNV disease incidence [65]. In urban landscapes in Chicago and Detroit, the WNF case rate was considerably higher in the urban class associated with the inner suburbs, where vegetation cover is moderate, and population density is moderate [10]. Deichmeister and Telang [74] concluded that urban infrastructure is positively correlated with the abundance of *Cx. pipiens* L./*Cx. restuans*. Their findings also implicated combined sewer overflow systems as large contributors to *Culex* vector populations.

Highly urbanized areas with diverse habitats can contribute to bird abundance [75] and constitute a certain vulnerability to WNV. Bertolotti *et al*. [11] found that birds in urban-vegetated areas and vegetative landscaping possessed a higher probability of virus propagation than those found in less vegetated areas. LaDeau *et al*. [2] showed that crows, like humans [65], have a higher exposure to WNV in more urban and less forested landscapes. Overall, the urban environment in which the WNF outbreaks occur is a diverse mix of buildings, transportation routes, vegetation, land uses and people associated with many aspects of urban life, from economic activity, to crime, to patterns of illness [10]. 

Mosquito species (e.g., *Cx. tarsalis*) appear to be abundant in uniform habitats with lower elevations and warmer temperatures [76]. In their study in northern Colorado, Eisen *et al*. [76] found that mosquito species richness is highest in plains habitats at elevations below 1,600 m. Later, Liu and Weng [77] showed in southern California that areas with lower elevations tended to be more susceptible to WNV invasion. This finding may be explained by the higher landscape diversity usually associated with multiple land cover types such as urban, grass, and water.

The WNF life cycle and mosquito habitat are, in part, a function of vegetation characteristics. However, relationships between disease patterns and vegetation indices in a particular region are difficult to extrapolate to other areas. Vegetation opacity is a composite indicator of temperature and moisture conditions which potentially influence mosquito activity and larval habitats. For instance, Chuang *et al*. [78] demonstrated that vegetation opacity and temperature are the two most important factors for predicting population numbers for *Cx. tarsalis*. Several studies have used vegetation indices as biological indicators of vector activity or disease incidence [79,80]. While Bowden *et al*. [81] demonstrated varying regional relationships between land cover and WNV incidence, strong associations were found between irrigated land and disease occurrence in the United States [82]. Similar with any other standing water bodies, irrigated lands serve as breeding habitats for mosquito populations and as a result, increase the risk of disease transmission. Monitoring of the first WNV epidemic in Spain (between 2010 and 2011) showed that many wetlands support large bird populations. The plenty of competent vectors indicate that ideal conditions for creating endemic cycles and WNV re-introduction are existed in Andalusia (Spain) [83].

### 2.3. Avian Hosts

WNV is transmitted primarily between avian hosts and mosquito vectors. Data from the Old World indicate that ideal conditions for WNV combine three key factors: a viremic, infectious host bird as the primary vertebrate hosts; active, ornithophilic mosquitoes (e.g., *Cx. pipiens*), as the principal vectors; and large numbers of one or more amplifying avian host species [84]. Increased immunity in the resident avian population may have also prevented the re-establishment of an enzootic amplification cycle sufficient to cause significant human disease [85].

WNV outbreaks are driven by the ongoing presence of infected avian populations since elevated WNV viremia in avian hosts is critical for establishing infections in *Culex* mosquitoes. Bird species and populations may vary in their susceptibility to infection. Their competence is determined by the amplitude and duration of the viremia period and can be expressed in relation to vector susceptibility to infection [86]. A relationship exists between avian biodiversity and WNF in humans since increased biodiversity can moderate the disease risk [87,88,89]. Moreover, Bradley *et al*. [90] showed that human population density and urbanization degree are positively correlated with exposure of passerine birds to WNV. Another major feature of WNV spatial dissemination is the high velocity of geographic invasion and colonization due to the long distance flight of birds, and to the ubiquitous presence of mosquitoes [91]. Bird migration influences new areas of spread. Liu *et al*. [92] suggest that the spatial spread of WNV is largely determined by the long-range dispersal of birds. Their patchy model simulations illustrated that discontinuous spatial spread arises very naturally as a consequence of local interaction between birds and mosquitoes, short-distance diffusion of mosquitoes, and long-range jump dispersal of birds. Maidana and Yang [91] used epidemiological modeling and simulations and introduced bird movements to understand the basic features of spatially distributed WNV from New York to California in five years. They showed that mosquito movements do not play an important role in disease dissemination, while bird movement becomes an important factor for lower mosquito biting rates.

The introduction of WNV to Europe is hypothesized to be mediated by migratory birds, which may be infected in Africa, and then carry the virus northward during spring migrations to European locations. [15,93,94]. This hypothesis, is based on serological findings in migratory birds (e.g., [95,96]), and aims to explain the geographic dispersal of the virus, and the timing of outbreaks that coincide when migrant birds carrying the virus northward during spring migration. It also describes why outbreaks often occur in or near wetlands and urban areas, where migratory bird populations, vectors and amplifying hosts are presented together [15,93]. The role of migratory birds in WNV dispersion into Europe has been assessed by several studies for Western Europe [96,97,98,99,100,101,102,103], for Eastern Europe [104,105], and for the Middle East [95]. Migratory birds are important in spreading the virus, as with the introduction of WNV into the eastern Mediterranean leading to multiple genotypes circulating concurrently [106]. Western Mediterranean wetlands such as the Camargue region (Southern France) attract birds from central Asia, Siberia, Northern and Eastern Europe, Western Africa, as well as the Mediterranean basin, and numerous birds of various species are seasonally aggregated in these habitats [107].

Although the ability of WNV to spread quickly along migration routes has been demonstrated, the precise mechanism of virus spread by migrating birds is still unknown and requires further research [25,108,109]. Nevertheless, a comparison between bird migration tracks during spring and WNF outbreaks in Europe and neighboring countries showed a very clear linkage between migration tracks and outbreak areas in Israel, Turkey, Romania, Russia, Italy, and Spain (human cases) and in Morocco, Gibraltar, and Italy for equine morbidity, but less apparent for Greece [28].

Long-distance migratory birds are not the only way to explain the spread of WNV over large distances; the virus could have been moved shorter distances sequentially by multiple individuals of short- and long-distance migratory species [93]. Wind patterns may also contribute to virus spread by wind-blown mosquitoes [110]. For example, it was found that wind is used by *Cx. tritaeniorhynchus* mosquitoes as a means of migration in China [111]. An alternative mechanism of virus spread is through dispersal from an endemic area via the movement of infected mosquitoes [112]. Tsuda *et al*. [113] estimated the maximum flight distance of host-seeking *Cx. pipiens pallens* as 1.2 km. This was nearly equal to that of *Cx. pipiens quinquefasciatus*. Interesting results were obtained also by Walton *et al*. [114] for *Cx. erythrothorax* in mark-recapture studies conducted in California, who showed that the daily mean distance traveled averaged 1.5, 0.46, and 0.50 km per night for August, September and October, respectively.

### 2.4. Other Drivers

In a world that is ever more interconnected through rapid transportation systems, such as passenger air travel and global trade, the virus is now being dispersed internationally. A large number of other drivers may contribute to the geographic spread of WNV. International travel, tourism and trade play an important role in the worldwide dispersion of pathogens and invasive vector species, at a staggering economic price [115,116]. Bird migration or trade, mosquito transportation by shipping, airplanes, or wind, and the movement of people are some of the factors that have impacts on the shifting distribution of WNV. Geographic distance to other infected areas may influence local WNV prevalence and rate of spread [64].

It can be expected that epidemiology of WNV-induced disease in people will vary between regions as a result of differences at the socio-demographic level. Populations living in poverty might be less likely to have secure, air-conditioned homes, and thus are more exposed to biting mosquitoes [55,117]. For example, Tackett *et al*. [118] found that poverty rates in thirteen states throughout the U.S. were related to the probability of a fatal outcome of WNF.

## 3. Discussion

Abiotic and biotic environmental factors have both contributed to the endemization of WNF in Europe during the past decades. These include favorable climatic conditions (such as ambient temperature, humidity, rainfall); hospitable habitats for high densities of competent mosquitoes (*Cx pipiens pipiens*, *Cx. modestus*, *Mansonia richiardii*); infected migratory birds for the dispersion of WNV; local birds for WNV amplification; presence of competent bridge vectors (such as *Cx. pipiens molestus*, *Aedes* spp.) for the transmission to humans; susceptible human and equine populations. The mosquito population density is a function of a number of environmental determinants such as elevated ambient temperatures, extreme rain events with flooding followed by dry and warm weather [119]. It also depends on presence of appropriate aquatic habitat for immature stages of the mosquito, such including irrigated land or flooded basements.

In light of the complex nature of all these different drivers involved in WNV transmission cycles in Europe, including a large number of mosquito vector and vertebrate species, the impact of future climate change will be difficult to quantify. Nevertheless, elevated temperatures due to climate change could increase mosquito population densities, shift the patterns of seasonal mosquito activity, and accelerate the reproduction rate of WNV in these vectors as discussed. Therefore, it is very possible that climate change will have a significant bearing on WNV transmission, exemplified by climate variability events such as the 2010 heat wave in Southeast Europe in 2010.

It is worth noting that global warming may also indirectly impact WNV transmission. Rappole and Hubálek [93] proposed three possible scenarios in which birds could serve as the introductory host for WNV into the New World: normal migration, storm-driven birds and importation. Several studies have shown that birds are migrating to their breeding grounds earlier in recent years, largely due to increases in average global temperatures [120,121,122,123] and in particular to the rise of mean spring temperatures [124]. Climatic changes seem to have complex and opposing effects on the timing of subsequent events in the annual cycle: depending on the ecology and life history of a species, as a reaction to increasing spring temperature in recent years, populations of migratory birds have advanced the seasonal start of their reproduction. Migratory bird species show a various amount of variation in their migratory responses [125,126]. These changes in migration timing may have an impact on WNV transmission but, to the best of our knowledge, have not yet been studied.

Regional changes in the location and intensity of storm tracks over the oceans can also affect bird migration across the Atlantic [127]. Indeed, an unprecedented change since 1950 was found in atmospheric, oceanic, and ecological indices describing North Atlantic climate variability [128]. Webster *et al*. [129] examined the number of tropical cyclones and cyclone days as well as tropical cyclone intensity over 35 years, in an environment of sea surface temperature increase. Although the smallest increase of severe hurricanes occurred in the North Atlantic Ocean, the number of cyclones and cyclone days has significantly increased. In light of these findings, it seems that change in tropical storms may influence WNV dispersal, by impacting the dynamics of storm-driven birds.

## 4. Conclusions

WNF is a serious public health issue, particularly in certain areas of Europe and increasingly so under climate change scenarios [130]. Reduced human exposure to mosquitoes infected with WNV, through vector abatement measures, is currently the most effective way to prevent transmission. Mosquito control efforts are also crucial to control an epidemic once transmission has begun; however, ideally, steps for mosquito abatement should be initiated before disease transmission to humans and domestic animals has occurred, through larval control measures. Ecologically based land-use planning, combined with improved development and sanitation, could reduce contact with and the abundance of human-commensal species and hence transmission of their pathogens [131]. Ambient temperature can be a determinant of WNF outbreaks, as discussed here, and it might be possible to initiate targeted larval abatement efforts immediately after significant temperature deviations have been detected in high risk areas. A critical component of such a vector control program is concurrent public education and health promotion to prevent and reduce risk of exposure. Understanding the environmental drivers of the WNF epidemiology in Europe will help guide these public health efforts.
